# Effect of DNA Origami Nanostructures on hIAPP Aggregation

**DOI:** 10.3390/nano10112200

**Published:** 2020-11-04

**Authors:** Marcel Hanke, Alejandro Gonzalez Orive, Guido Grundmeier, Adrian Keller

**Affiliations:** 1 Technical and Macromolecular Chemistry, Paderborn University, Warburger Str. 100, 33098 Paderborn, Germany; marcelha@mail.uni-paderborn.de (M.H.); agorive@ull.edu.es (A.G.O.); g.grundmeier@tc.uni-paderborn.de (G.G.); 2 Department of Chemistry, University of La Laguna, P.O. Box 456, E-38200 La Laguna, Spain

**Keywords:** DNA origami, self-assembly, peptides, amyloid, atomic force microscopy

## Abstract

The aggregation of human islet amyloid polypeptide (hIAPP) plays a major role in the pathogenesis of type 2 diabetes mellitus (T2DM), and numerous strategies for controlling hIAPP aggregation have been investigated so far. In particular, several organic and inorganic nanoparticles (NPs) have shown the potential to influence the aggregation of hIAPP and other amyloidogenic proteins and peptides. In addition to conventional NPs, DNA nanostructures are receiving more and more attention from the biomedical field. Therefore, in this work, we investigated the effects of two different DNA origami nanostructures on hIAPP aggregation. To this end, we employed in situ turbidity measurements and ex situ atomic force microscopy (AFM). The turbidity measurements revealed a retarding effect of the DNA nanostructures on hIAPP aggregation, while the AFM results showed the co-aggregation of hIAPP with the DNA origami nanostructures into hybrid peptide–DNA aggregates. We assume that this was caused by strong electrostatic interactions between the negatively charged DNA origami nanostructures and the positively charged peptide. Most intriguingly, the influence of the DNA origami nanostructures on hIAPP aggregation differed from that of genomic double-stranded DNA (dsDNA) and appeared to depend on DNA origami superstructure. DNA origami nanostructures may thus represent a novel route for modulating amyloid aggregation in vivo.

## 1. Introduction

The assembly of soluble protein and (poly)peptide monomers into β-sheet-rich oligomeric and fibrillar amyloid aggregates is not only a fascinating and highly complex phenomenon [1], but also of extraordinary relevance in biology and medicine. While there are many functional amyloids deliberately produced by various organisms [2,3], amyloid aggregation also represents a common link in many degenerative diseases including Alzheimer’s disease, Parkinson’s disease, and type 2 diabetes mellitus (T2DM) [4]. During the course of such so-called misfolding diseases, cytotoxic amyloid aggregates assembled from various proteins and peptides appear in different organs and slowly replace healthy tissue [5]. For instance, the pathogeneses of T2DM involves the aggregation of human islet amyloid polypeptide (hIAPP), a positively charged 37-residue polypeptide hormone co-located and co-secreted with insulin in the β cells of the pancreas [6]. The formation of oligomeric hIAPP as well as the growth of fibrils on the cell membranes of pancreatic β cells may lead to membrane damage and thus to β-cell death [7].

Despite their great molecular diversity, amyloid fibrils assembled from different molecules are surprisingly similar in morphology and molecular structure. The latter in particular is characterized by an intermolecular cross-β structure that features β-strands oriented perpendicular to the fibril axis [8]. Nevertheless, amyloid aggregation turned out to be a highly complex process that is strongly dependent not only on the molecular species involved but also on numerous environmental parameters such as temperature, monomer concentration, electrolyte composition, and the presence of interfaces [9]. Consequently, understanding and ultimately controlling the molecular mechanisms that govern amyloid aggregation in vivo have proven rather difficult indeed [10,11].

The advent and rise of biomedical nanotechnology have spawned a plethora of therapeutic and diagnostic nanoparticle (NP) systems, several of which have received FDA approval and are now routinely employed in clinical practice [12]. Upon interaction with physiological fluids, NPs develop a highly dynamic protein corona [13]. Since NP–protein interactions often result in conformational changes in the interacting proteins [13], they may also affect the proteins’ propensity to form amyloid aggregates. Indeed, many organic and inorganic NP systems were found to be able to inhibit or promote the aggregation of disease-relevant proteins and peptides [9,14,15]. While this may result in unwanted side effects of therapeutic and diagnostic NPs, it may also provide a novel NP-based approach for treating amyloid diseases [16].

A rather novel class of biomedical NP systems with great therapeutic and diagnostic potential comprises DNA nanostructures [17,18,19]. DNA self-assembly and in particular DNA origami technology [20] enable the high-yield synthesis of fully biocompatible, biodegradable, and non-cytotoxic nanostructures with almost arbitrary shapes, that can be employed as delivery vehicles for various therapeutic cargos such as antibodies, enzymes, nucleic acids, and NPs [17,18,19]. Furthermore, their surfaces can be modified with functional entities such as aptamers to facilitate cell targeting, cellular uptake, and cargo release [17,18,19]. A number of recent studies have demonstrated successful in vivo treatment of various diseases, including different tumors [21,22,23,24], acute kidney injury [25], and cerebral ischemia–reperfusion injury [26]. However, the effect of DNA nanostructures on the aggregation of medically relevant amyloidogenic peptides and proteins has not yet been studied.

In this work, we thus investigated the interaction of two different DNA origami nanostructures with the T2DM-associated peptide hIAPP. The combination of these two molecular species is particularly interesting because DNA origami nanostructures are strongly negatively charged, while the aggregation of positively charged hIAPP is known to be strongly affected by its interaction with negatively charged surfaces [27]. The initial stages of hIAPP aggregation were investigated in situ by turbidity measurements, whereas ex situ atomic force microscopy (AFM) was used to characterize aggregate morphology. We found that the presence of DNA origami nanostructures slowed down the kinetics of hIAPP aggregation via a strong attractive interaction between the amyloid aggregates and the DNA nanostructures. The effects of the DNA origami nanostructures on hIAPP aggregation differed from those of genomic double-stranded DNA (dsDNA). DNA origami nanostructures may thus present a novel approach toward controlling amyloid aggregation.

## 2. Materials and Methods

### 2.1. hIAPP Preparation

For denaturation and purification of the peptide, 1 mg of hIAPP (BACHEM AG, Bubendorf, Switzerland) was dissolved in 512 µL 1,1,1,3,3,3-hexafluoro-2-propanol (HFIP, Thermo Fisher GmbH, Kandel, Germany) and stored for 1 h at room temperature with occasional vortexing. Then, the peptide solution was centrifuged (15,000 rpm) at 4 °C for 30 min using a microcentrifuge (VWR International GmbH, Darmstadt, Germany). In the next preparation step, the upper ~80% of the solution was divided into 20 µL aliquots and left in the fume hood overnight in order to evaporate the HFIP and obtain a dried peptide film. The dried aliquots were stored at −20 °C. For concentration adjustment, the dry peptide films were redissolved in HFIP, vortexed for 30 s, centrifuged for 15 s, using an Eppendorf MiniSpin centrifuge (Eppendorf AG, Hamburg, Germany) and subjected to the same treatment as described above.

### 2.2. DNA Origami and Genomic dsDNA Preparation

For the preparation of DNA origami triangles [20] and six-helix bundles (6HBs) [28], the 7249 nt M13mp18 scaffold and about 200 staple strands (Metabion international AG, Planegg/Steinkirchen, Germany) were annealed at a molar ratio of 1:10 in 1 × TAE buffer (Carl Roth GmbH + Co. KG, Karlsruhe, Germany) supplemented with 10 mM MgCl_2_ (Sigma-Aldrich Chemie GmbH, Steinheim, Germany) by gradually decreasing the temperature from 80 °C to RT over 1.5 h in a Primus 25 advanced thermocycler (PEQLAB, Erlangen, Germany). The samples were purified using Amicon Ultra-0.5 mL spin filters with 100 kDa molecular weight cut-off (Merck KGaA, Darmstadt, Germany). The concentrations of the obtained DNA origami solutions were determined using an Implen Nanophotometer P330 (Implen GmbH, München, Germany) and adjusted to the desired values.

Genomic dsDNA from salmon testes (Thermo Fisher GmbH, Kandel, Germany) was used as a control with similar GC content as the assembled DNA origami nanostructures [29,30] but no defined length or secondary structure, in order to distinguish between duplex- and superstructure-specific effects. It was dissolved in 1 × TAE buffer with 10 mM MgCl_2_ and the concentration (in bp) adjusted using an Implen Nanophotometer P330 (Implen, München, German).

### 2.3. Sample Preparation

Before each experiment, one hIAPP aliquot was slowly brought to room temperature, dissolved in 100 µL DMSO (Sigma-Aldrich Chemie GmbH, Steinheim, Germany), and allowed to reach equilibrium for 15 min. The hIAPP–DMSO solution was added to 900 µL of 1.1 × TAE buffer, containing 10 mM MgCl_2_ (either pure or with the respective DNA sample) and resulting mixture vortexed for 15 s. The final samples had concentrations of 1 µM hIAPP, 10% DMSO, and 36.25 µM of DNA bp, respectively.

### 2.4. Turbidity Measurements

Turbidity measurements were performed using an Implen Nanophotometer P330 with a 1 mL quartz cuvette (Hellma GmbH & Co. KG, Müllheim, Germany). A blank of pure 1 × TAE buffer with 10 mM MgCl_2_ was used. The absorption of the freshly prepared samples at 600 nm was measured three times at each time point. Between measurements, the cuvette with the sample was incubated at 37 °C without shaking.

### 2.5. AFM

For the AFM-based characterization of the hIAPP aggregates, the 1 mL sample solutions were incubated at 37 °C in a quartz cuvette (Hellma GmbH & Co. KG, Müllheim, Germany) without shaking. At certain time points, the sample solutions were gently mixed with a 100 µL pipette three times in order to also retrieve segregated aggregates. Then, 30 µL of each sample were removed and deposited onto freshly cleaved mica, incubated for 15 min, immersed in HPLC-grade water (VWR International S.A.S., Fontenay-sous-Bois, France) for 30 s, and blow-dried with ultra-pure air. AFM imaging was performed using a JPK Nanowizard II and a JPK Nanowizard III AFM (JPK Instruments AG, Berlin, Germany) operated in intermittent contact mode in air with HQ:NSC18/Al BS cantilevers (75 kHz and 2.8 N/m) from MikroMasch (NanoAndMore GmbH, Wetzlar, Germany).

For the characterization of the DNA origami nanostructures without exposure to hIAPP, two solutions of each DNA nanostructure (5 nM) with and without 10% of DMSO were prepared. Prior to AFM imaging, the DMSO-containing solutions (100 µL) each were incubated for 1 h at 37 °C to mimic the incubation conditions of the hIAPP samples. For AFM imaging, 1 µL of each sample was deposited on freshly cleaved mica, immediately covered with 50 µL of 1 × TAE buffer with 10 mM MgCl_2_, and incubated for 3 min. After incubation, the samples were washed with HPLC-grade water (VWR International S.A.S., Fontenay-sous-Bois, France) and dried in ultra-pure air.

The recorded AFM images were analyzed using Gwyddion open source software version 2.56 [31]. Fibril heights were measured manually from height profiles. Aggregate volumes and boundary lengths were determined by applying a height threshold to the flattened images using the Mark by Threshold tool. The value of the threshold was adjusted individually to exclude the mica surface and the background DNA while masking larger aggregates such as fibrils and fibril clusters (see Figures S2–S13, Supplementary Materials). The zero-basis volumes and projected boundary lengths of all the masked areas were then extracted using the Grain Distributions tool.

## 3. Results

**Figure 1:**
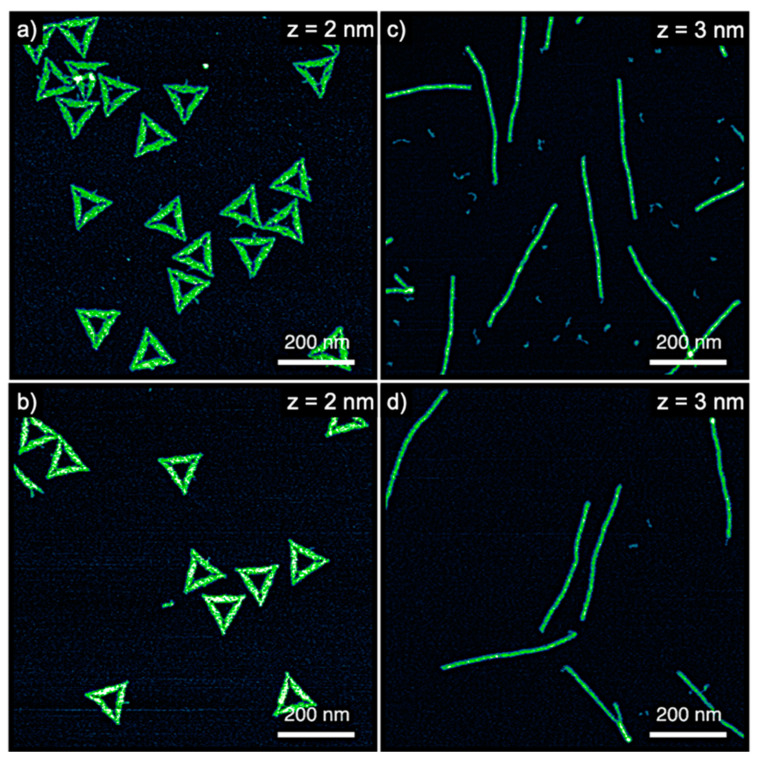
Atomic force microscopy (AFM) images of (**a**,**b**) DNA origami triangles and (**c**,**d**) 6HBs incubated in buffer (**b**,**d**) with and (**a**,**b**) without 10% DMSO. The ranges of the z-scales are given in the individual images.

To study the effect of DNA origami nanostructures on hIAPP aggregation, we selected two different DNA origami shapes, i.e., the Rothemund triangle [20] and a six-helix bundle (6HB) [28]. AFM images of both structures are shown in Figure 1a,c. Before mixing the DNA origami with the hIAPP samples, however, we tested their stability in the final buffer composition. Many established protocols for the preparation of monomeric starting solutions of hIAPP [27,32] and other amyloidogenic peptides [33,34] free of any preformed aggregates involve the dissolution of the peptide sample in dimethyl sulfoxide (DMSO) at final concentrations typically between 1% and 10%. However, DMSO is a known DNA denaturant [35], while DNA origami nanostructures are often observed to be more sensitive toward chemical denaturation than normal dsDNA [36]. Nevertheless, as the AFM images in Figure 1b,d demonstrate, both the DNA origami triangles and 6HBs do not show any structural damage in 10% DMSO-containing buffer after 1 h incubation at 37 °C.

Next, we set out to study the effect of the different DNA structures on the aggregation kinetics of hIAPP in situ. This is usually done using the well-established thioflavin T (ThT) fluorescence assay [37,38]. However, since ThT also binds to DNA, which may result in drastically enhanced fluorescence (see Figure S1, Supplementary Materials) [39,40,41], we turned to turbidity measurements instead in order to characterize hIAPP aggregation kinetics in the presence of the different DNA structures [42,43,44].

**Figure 2:**
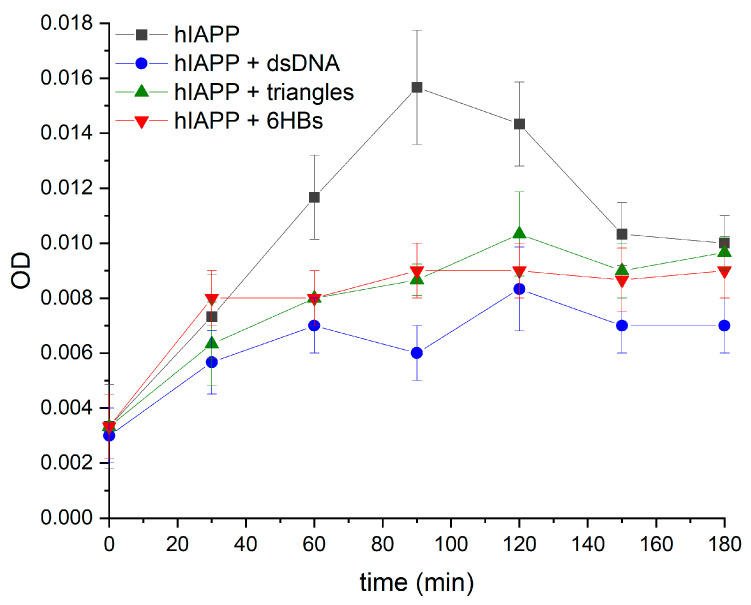
Optical density (OD) at 600 nm of human islet amyloid polypeptide (hIAPP) with and without different DNA structures as a function of incubation time.

As can be seen in Figure 2, the optical density (OD) for pure hIAPP is monotonically increasing with incubation time and reaches a maximum after 90 min. For longer incubation times, the OD decreases again, which can be attributed to a precipitation of large, insoluble hIAPP aggregates in the cuvette. Indeed, for incubation times beyond 3 h, precipitation was so strong that it could be noticed by the naked eye. However, in the presence of dsDNA and DNA origami nanostructures, the OD saturates earlier and at a lower level. After an initial increase, the OD increases only very mildly for incubation times longer than 30 min. From this point on, the OD is much lower than for pure hIAPP. After 120 min, the DNA-containing samples also exhibit a slight decrease in OD, presumably due to similar precipitation effects. In contrast to pure hIAPP, however, no macroscopic precipitation could be observed after incubation for several hours. As an interesting observation, samples with the DNA origami triangles and 6HBs show almost identical behavior in the turbidity measurements, whereas the hIAPP sample with dsDNA shows a slightly lower OD at all incubation times. This may hint at DNA structure-specific differences in their effect on hIAPP aggregation.

**Figure 3:**
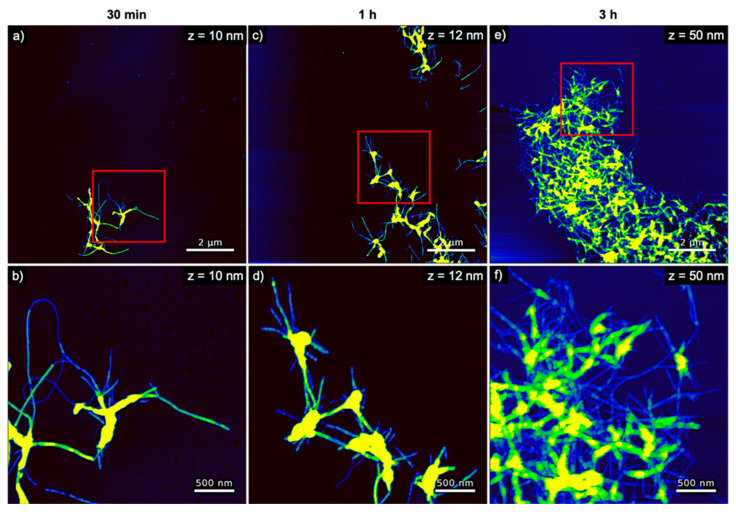
AFM images of pure hIAPP incubated without any DNA for (**a**,**b**) 30 min, (**c**,**d**) 1 h, and (**e**,**f**) 3 h. The images in (**b**,**d**,**f**) represent zooms of the regions indicated in the respective images in (**a**,**c**,**e**). The ranges of the z-scales are given in the individual images.

**Figure 4:**
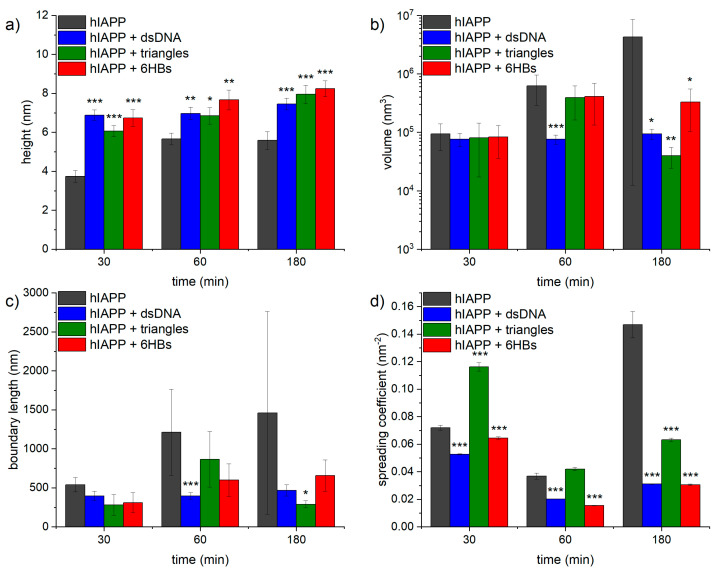
(**a**) Average heights of hIAPP fibrils obtained in the presence and absence of the different DNA structures. Values represent averages of 21 to 30 individual fibrils. (**b**) Average volumes of hIAPP aggregates obtained in the presence and absence of the different DNA structures. Values represent averages of 163 to 5285 individual aggregate structures. Note the logarithmic *y*-axis. (**c**) Average boundary lengths of hIAPP aggregates obtained in the presence and absence of the different DNA structures. Values represent averages of 163 to 5285 individual aggregate structures. (**d**) Average spreading coefficients of hIAPP aggregates obtained in the presence and absence of the different DNA structures. Values represent averages of 163 to 5285 individual aggregate structures. Error bars indicate the standard error of the mean. Significances (two-tailed distribution, homoscedastic) are given with respect to pure hIAPP at the respective incubation times and indicated as * (*p* < 0.05), ** (*p* < 0.01), and *** (*p* < 0.001).

The evolution of hIAPP aggregate morphology was assessed ex situ using time-lapse AFM. As can be seen in Figure 3, many comparatively short fibrils with heights of ~4 nm are observed after 30 min of incubation. With increasing incubation time, these fibrils seem to become longer and their height increases to about 6 nm (see Figure 4a). The fibrils are found to interact with each other and form differently sized bundles and clusters, which grow larger with increasing incubation time and reach dimensions of several 10 µm after 3 h incubation. Furthermore, and in accordance with the observed decrease of the OD at long incubation times, the AFM images show a strong variation in surface coverage for 1 and 3 h incubation (see Figures S2–S4), hinting at the precipitation of insoluble aggregates.

**Figure 5:**
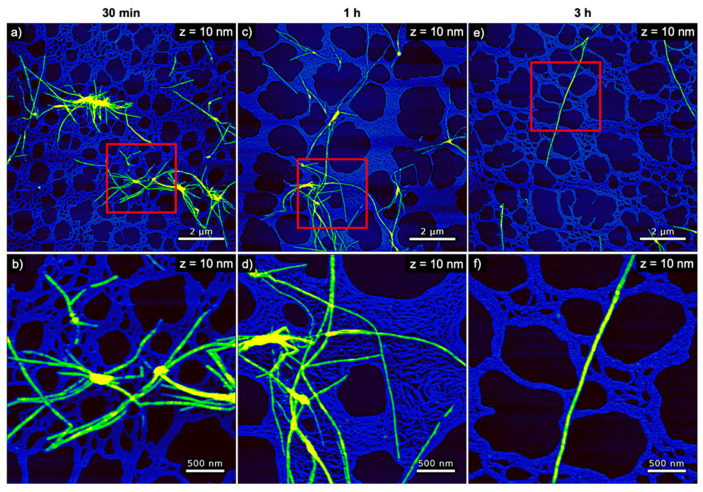
AFM images of hIAPP incubated with genomic dsDNA for (**a**,**b**) 30 min, (**c**,**d**) 1 h, and (**e**,**f**) 3 h. The images in (**b**,**d**,**f**) represent zooms of the regions indicated in the respective images in (**a**,**c**,**e**). The ranges of the z-scales are given in the individual image.

The AFM images of hIAPP incubated with genomic dsDNA are shown in Figure 5. Here, a percolated DNA network with a height of ~2 nm is always visible in the background of the images. Such percolated networks are frequently observed in DNA films adsorbed from concentrated DNA solutions [45,46,47,48,49,50,51,52]. Due to their larger heights of 7 nm, the hIAPP fibrils can be distinguished fairly well from this network. No change in fibril height is observed with increasing incubation time (see Figure 4a). Furthermore, the fibrils in general appear longer than those observed for pure hIAPP in Figure 3 and form clusters consisting of fewer fibrils. The latter may indicate that the hIAPP–DNA interaction reduces the tendency of the hIAPP fibrils to associate with one another, presumably by blocking attractive interactions. However, the AFM images in Figure 5 and Figures S5–S7 also show fewer amyloid aggregates on the mica surface after 3 h incubation than for shorter incubation times, which may again be indicative of the precipitation of larger aggregates, albeit at a lower extent than for pure hIAPP.

Interestingly, the entire population of amyloid fibrils observed in the AFM images is associated directly with the DNA network and not a single amyloid fibril is found in direct contact with the mica surface (see in particular the zoomed AFM images in Figure 5b,d,f). This indicates that the retarding effect of DNA results from strong electrostatic interactions between the positively charged hIAPP and the negatively charged phosphate groups of the DNA. This direct interaction of the hIAPP fibrils with the dsDNA may also be responsible for the larger fibril height compared to pure hIAPP (see Figure 4a). In particular, the average fibril height is increased by 1 to 3 nm, which agrees fairly well with the diameter of the DNA duplex of 2 nm.

In order to analyze the morphological differences between the hIAPP aggregates obtained in the presence and absences of DNA quantitatively, we measured the volume and boundary length of each aggregate structure observed in the AFM image (see Section 2.5. for details). This analysis included particle-like aggregates, individual fibrils, and large fibril bundles and clusters, while excluding the DNA visible in the background (see Figures S2–S13). As can be seen in Figure 4b, the average aggregate volume of pure hIAPP increases from about 10^5^ nm³ after 30 min incubation to about 4 × 10^6^ nm³ after incubation for 3 h. Furthermore, the variation of the aggregate volume as indicated by the error bars in Figure 4b is also increasing with time, reflecting the growing heterogeneity in aggregate size (see also Figures S2–S4). In the presence of dsDNA, however, the aggregate volume remains rather constant at slightly below 10^5^ nm³ with essentially identical error bars over the same time course. Similar trends are observed also for the boundary length shown in Figure 4c. The latter is not surprising as the boundary length of any object is of course related to its volume. In order to assess changes in the overall shape of the aggregates, we therefore defined a spreading coefficient, which relates the lateral spread of an aggregate to its volume by dividing the boundary length by the volume. Highly compact aggregates such as spherical particles thus have a small spreading coefficient, whereas fringed and rugged aggregates such as loosely intertwined fibrils have a large one. As can be seen in Figure 4d, the average spreading coefficient of the pure hIAPP aggregates shows a rather interesting behavior as an initial decrease between 30 and 60 min of incubation is followed by a strong increase between 60 and 180 min. This indicates that isolated fibrils are forming at short incubation times, which then start to interact with each other and form compact fibril bundles and clusters. These aggregate clusters then grow further into extended, highly porous networks of entangled fibrils (see Figure 3). A similar yet less pronounced trend is observed also in the presence of dsDNA (see Figure 4d). In this case, however, the spreading coefficient is always significantly below that of pure hIAPP, indicating that the aggregates are in general more compact. All these data thus clearly show an effect of dsDNA on the morphology of the hIAPP aggregates.

**Figure 6:**
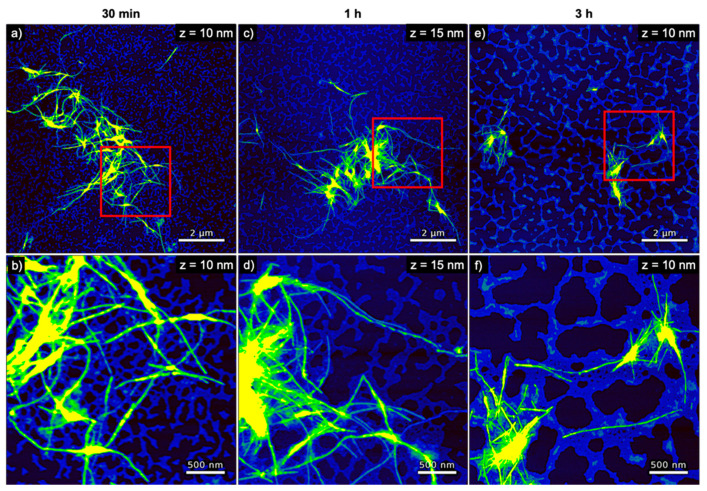
AFM images of hIAPP incubated with DNA origami triangles for (**a**,**b**) 30 min, (**c**,**d**) 1 h, and (**e**,**f**) 3 h. The images in (**b**,**d**,**f**) row represent zooms of the regions indicated in the respective images in (**a**,**c**,**e**). The ranges of the z-scales are given in the individual image.

Figure 6 shows AFM images of hIAPP incubated with DNA origami triangles. Due to the comparatively large DNA origami concentration and long incubation time on the mica substrate, the DNA origami triangles also form a dense yet percolated layer at the surface. The DNA origami appear largely intact but somewhat deformed and bulging (see Figures S8–S10), which may be attributed to the negatively charged DNA origami triangles being coated with the positively charged peptides. Furthermore, all amyloid fibrils are associated with DNA origami triangles, similar to the case of the genomic dsDNA in Figure 5. This is particularly apparent in the image in Figure 6f, where dense DNA origami halos can be observed around the fibril clusters and the single fibrils. This again hints at a high affinity of hIAPP for DNA due to attractive electrostatic interactions between the positively charged amino acid residues of the peptide and the negatively charged phosphate groups of the DNA. This strong electrostatic interaction may also be responsible for the formation of the dense DNA origami films observed at the mica surface, as hIAPP binding may lead to a partial charge inversion of the DNA origami and thus favor DNA origami aggregation. Furthermore, for long incubation times, this attractive interaction manifests in a notable depletion of DNA origami triangles on the mica surface with the remaining DNA origami appearing even more aggregated (see Figure 6e,f). The average height of the hIAPP fibrils is similar to the case of genomic dsDNA and exhibits a steady increase from ~6 nm after 30 min incubation to ~8 nm after 3 h (see Figure 4a).

With regard to the morphological parameters of the aggregates, however, certain differences are observed between the DNA origami triangles and genomic dsDNA. As can be seen in Figure 4b,c, both aggregate volume and boundary length are basically identical for the two DNA structures for incubation times of 30 min and 3 h. At an intermediate incubation time of 1 h, however, the aggregates in the presence of the DNA origami triangles have a higher average volume and boundary length than in the presence of dsDNA. For this particular incubation time, both parameters are in fact more similar to those of pure hIAPP. This may indicate that hIAPP aggregation and fibril cluster formation follow different kinetics in the presence of dsDNA and DNA origami triangles. Stronger differences are observed in the spreading coefficient shown in Figure 4d. Here, the values obtained in the presence of the DNA origami triangles are always larger than in the presence of dsDNA. More interestingly, however, the spreading coefficient for the DNA origami triangles after 30 min incubation is also much higher than for pure hIAPP. This situation is then reversing with time, so that after incubation for 3 h, the pure hIAPP aggregates have a higher average spreading coefficient than in the presence of DNA origami triangles.

**Figure 7:**
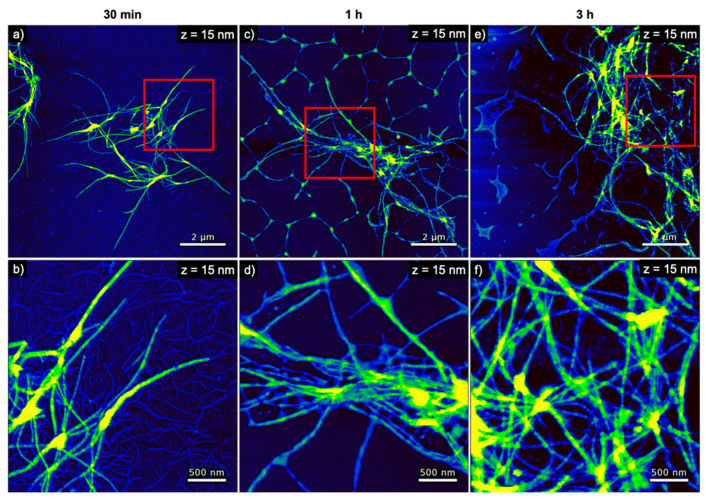
AFM images of hIAPP incubated with DNA origami 6HBs for (**a**,**b**) 30 min, (**c**,**d**) 1 h, and (**e**,**f**) 3 h. The images in (**b**,**d**,**f**) represent zooms of the regions indicated in the respective images in (**a**,**c**,**e**). The ranges of the z-scales are given in the individual images.

The AFM images of hIAPP samples incubated with DNA origami 6HBs shown in Figure 7 reveal a more pronounced formation of hIAPP fibrils. These fibrils again appear rather long and have a height of about 7 to 8 nm (see Figure 4a). In striking contrast to hIAPP aggregation in the presence of DNA origami triangles, large, network-like bundles of long, loosely associated fibrils are observed already after 30 min of incubation, which subsequently grow into extended networks of entangled fibrils that may spread over length scales of several 10 µm. After 30 min incubation, a dense layer of well-defined DNA origami 6HBs are observed on the mica surface and in particular surrounding the hIAPP fibrils (see Figure 7a,b). For longer incubation times of 1 h and 3 h, however, barely any individual 6HBs can be identified on the mica surface (see Figure 7c–f), suggesting that most of the 6HBs seem to have associated with and been incorporated into the fibril networks (see in particular Figure 7d,f). From a morphological point of view, the DNA origami 6HBs behave rather similarly to the other two DNA structures studied in this work, with only minor deviations in aggregate volume or boundary length. Perhaps somewhat surprisingly in view of the turbidity results shown in Figure 2, however, the average spreading coefficients of the hIAPP aggregates obtained in the presence of the 6HBs are almost identical to those of dsDNA, whereas for the DNA origami triangles considerably larger values were observed. In contrast, the turbidity measurements in Figure 2 revealed almost identical OD values for the two DNA origami nanostructures, whereas the ones determined in the presence of genomic dsDNA were slightly lower.

## 4. Discussion

Amyloid aggregation follows complex pathways that may involve numerous different intermediate species, i.e., intact and misfolded monomers, amorphous aggregates, β-sheet-stabilized oligomers, protofibrils, mature fibrils, and fibril bundles and clusters [53]. Disturbing the relative concentration or formation rate of one or more of these species may thus result in pronounced and often complex variations in the overall aggregation kinetics and the morphological and structural properties of intermediate and final aggregates. Depending on their physicochemical properties, NPs may adsorb monomers, oligomers, and fibrils, thereby affecting the bulk concentrations of the different species and even inducing conformational changes in the adsorbed molecules. Each of these mechanisms may affect the pathway of aggregation and even lead to a switching from one pathway to another [54]. As we showed in this work, the presence of DNA in solution appears to have a somewhat retarding effect on the aggregation of hIAPP, presumably because of the electrostatic attraction between the negatively charged DNA backbone and the positively charged peptide. Interestingly, the shape of the DNA structures, i.e., genomic dsDNA, planar DNA origami triangles, and tube-like 6HBs, did not have a strong effect on the overall retardation of hIAPP aggregation. In contrast, we observed DNA structure-specific effects on the morphology of the assembled amyloid fibrils and in particular fibril clusters, which indicates that the presence of DNA not only affects hIAPP fibrillization but also modulates fibril–fibril interactions. Similar observations have recently been reported for the effect of differently shaped gold NPs on the aggregation of an amyloid-β peptide [55].

The experimental results presented above are further indicative of the co-aggregation of hIAPP and the different DNA structures into hybrid peptide–DNA fibrils and networks driven by the attractive electrostatic interactions. The electrostatic coating of DNA origami nanostructures with positively charged peptides [56,57,58], polyelectrolytes [59,60], and (modified) proteins [61,62,63] has frequently been reported in the literature. Furthermore, the aggregation of DNA nanostructures and other well-defined nanomaterials in complex mixtures has also been observed previously. Jiang et al. reported the co-assembly of various DNA origami structures and positively charged collagen-mimetic peptides into hybrid nanostructures in which the sheet-like DNA origami were stacked onto each other by intermediate peptide layers [64]. Electrostatics-driven aggregation of negatively charged actin filaments into bundles and networks in the presence of mono- and divalent cations was reported by Huber et al. [65]. Depending on the employed actin and cation concentrations, the authors observed the formation of different aggregates, including aster-like networks composed of star-shaped clusters of actin bundles. Similar aster-like networks were also observed for the condensation of actin filaments [66] and tile-based DNA nanotubes [67] by depletion forces. These aster-like networks are remarkably similar in appearance to the extended networks observed in the present work for the co-aggregation of hIAPP with the DNA origami 6HBs (see Figure 7 and Figures S11–S13). In contrast, the hybrid hIAPP–DNA aggregates observed in the presence of genomic DNA (see Figure 5 and Figures S5–S7) and DNA origami triangles (see Figure 6 and Figures S8–S10) rather resemble the previously described isolated bundles and isolated asters, respectively [66].

Different mechanisms may be involved in the formation of the different aggregate morphologies in the presence of the different DNA structures. Because of the electrostatic nature of the hIAPP–DNA interaction, different surface charge densities and backbone accessibilities of the genomic dsDNA and the different DNA origami nanostructures, which are assembled on the square and honeycomb lattice, respectively, may result in the modulation of interaction strength. Similar superstructure-dependent effects have previously been observed for the interaction of DNA origami nanostructures with multivalent cations [29], groove binders [30], and proteins [68,69,70].

However, the very different shapes of the DNA structures may also play a role in co-aggregation. The DNA origami 6HBs have a nominal length and solution diameter of 412 and 6 nm, respectively [28], and are thus geometrically very similar to the native hIAPP fibrils (see Figure 4a). They may thus be more efficiently integrated into a growing fibril or a forming fibril bundle than, for instance, the 2D DNA origami triangles. A similar effect has been observed in the electrostatics-driven assembly of 3D nanoparticle superlattices mediated by DNA origami helix bundles, in which 6HBs turned out to be more efficient than 24HBs and 60HBs in facilitating the formation of crystal-like co-aggregates [71]. The genomic dsDNA employed in this work has a diameter and average length of 2 nm and a few hundred nm [72], respectively, and is thus similarly anisotropic. However, the persistence length of dsDNA is about 50 nm [73], whereas that of DNA 6HBs was determined to be larger than 3 µm [66]. Therefore, it appears reasonable that the more flexible dsDNA is also more efficient in neutralizing or inverting the positive charges of the growing or grown peptide fibrils than the more rigid DNA origami 6HBs, which should result in a lower propensity to form extended bundles as observed in Figure 5 and Figures S5–S7. The more rod-like 6HBs, however, can only align along the growing or grown fibrils and due to their comparatively large bulk and surface charge density promote the association of individual fibrils. Indeed, while numerous studies have observed the formation of aster-, star-, or spindle-like aggregates of nanostructures from complex solutions, all these studies used highly anisotropic and mechanically rigid nanostructures such as the already mentioned DNA nanostructures [67,74] and actin filaments [65,66,75,76], but also microtubules [77,78] and phages [79]. It thus appears rather likely that shape anisotropy and mechanical properties of the employed DNA origami nanostructures play an important role in modulating hIAPP–DNA co-aggregation.

## 5. Conclusions

In this work, the effect of genomic dsDNA and two different DNA origami nanostructures on the aggregation of hIAPP was studied in situ and ex situ by turbidity measurements and AFM imaging, respectively. The presence of DNA was observed to affect hIAPP aggregation via a direct interaction of the positively charged peptide with the negatively charged DNA structures. The latter apparently became attached to or incorporated into the assembled amyloid fibrils, as indicated by the observation that virtually all hIAPP fibrils visible in the recorded AFM images were surrounded by a halo of DNA.

Distinct differences in the effect of the different DNA structures investigated in this work on hIAPP aggregation were observed. In particular, we found that 2D DNA origami triangles induced the formation hybrid hIAPP–DNA aggregates reminiscent of isolated asters, whereas isolated bundles of longer fibrils were observed in the presence of genomic dsDNA of similar GC content. In contrast, DNA origami 6HBs showed a more pronounced association with the formed hIAPP fibrils, resulting in large hybrid aster-like networks composed of 6HBs and amyloid fibrils. For long incubation times, the formation of these large networks even resulted in the depletion of free 6HBs in solution. These different aggregate morphologies are probably related to different mechanical and surface properties of the employed DNA structures, as well as to geometric anisotropy of the individual components. This may offer the intriguing possibility of modulating amyloid aggregation in vitro and possibly in vivo by employing specifically designed DNA origami nanostructures.

Despite the morphological differences between the hybrid hIAPP–DNA aggregates, the in situ turbidity measurements showed only little effect of the type of DNA structure on their overall effect on hIAPP aggregation. In particular, there was no discernible difference between the DNA origami triangles and the 6HBs, whereas slightly lower OD values were observed for genomic dsDNA. Even though this indicates that linear dsDNA is at least as efficient in retarding hIAPP aggregation as the more complex DNA origami nanostructures, the latter have greater therapeutic potential. On the one hand, untreated dsDNA is rapidly degraded by the body within minutes after injection [80] and thus typically requires the encapsulation in lipid, polymer, or viral vectors in order to serve as an efficient therapeutic agent [81]. DNA nanostructures, on the other hand, are significantly more stable under physiological conditions than dsDNA and thus have already been successfully employed in several in vivo studies [17]. In this context, our study also highlights the complexity of the DNA nanostructure–amyloid interaction, which may result in numerous negative side effects in the in vivo application of biomedical DNA nanostructures that have not yet been considered. These issues thus need to be investigated in detail for various DNA nanostructures and amyloidogenic proteins and peptides under relevant environmental conditions to ensure the safety of the numerous DNA nanostructure-based therapies currently in development.

## Supplementary Materials

The following are available online at https://www.mdpi.com/2079-4991/10/11/2200/s1, Figure S1: ThT fluorescence spectra in the absence and presence of dsDNA, Figure S2: AFM images of pure hIAPP incubated without any DNA for 30 min, Figure S3: AFM images of pure hIAPP incubated without any DNA for 1 h, Figure S4: AFM images of pure hIAPP incubated without any DNA for 3 h, Figure S5: AFM images of hIAPP incubated with genomic dsDNA for 30 min, Figure S6: AFM images of hIAPP incubated with genomic dsDNA for 1 h, Figure S7: AFM images of hIAPP incubated with genomic dsDNA for 3 h, Figure S8: AFM images of hIAPP incubated with DNA origami triangles for 30 min, Figure S9: AFM images of hIAPP incubated with DNA origami triangles for 1 h, Figure S10: AFM images of hIAPP incubated with DNA origami triangles for 3 h, Figure S11: AFM images of hIAPP incubated with DNA origami 6HBs for 30 min, Figure S12: AFM images of hIAPP incubated with DNA origami 6HBs for 1 h, Figure S13: AFM images of hIAPP incubated with DNA origami 6HBs for 3 h.
