# Supplementary material for: Effect of DNA Origami Nanostructures on hIAPP Aggregation
Source: Nanomaterials (Basel). 2020 Nov 4;10(11):2200. doi: 10.3390/nano10112200 (PMC7694230; doi:10.3390/nano10112200)
Supplement: Supplementary file 1 [file nanomaterials-10-02200-s001.pdf]

Supplementary Materials

Effect of DNA Origami Nanostructures on hIAPP Aggregation

Marcel Hanke [1], Alejandro Gonzalez Orive [1,2], Guido Grundmeier [1] and Adrian Keller [1,]*

[1] Technical and Macromolecular Chemistry, Paderborn University, Warburger Str. 100, 33098 Paderborn, Germany. *E-mail: adrian.keller@uni-paderborn.de

[2] Department of Chemistry, University of La Laguna, P.O. Box 456, E-38200 La Laguna, Tenerife, Canary Islands, Spain.

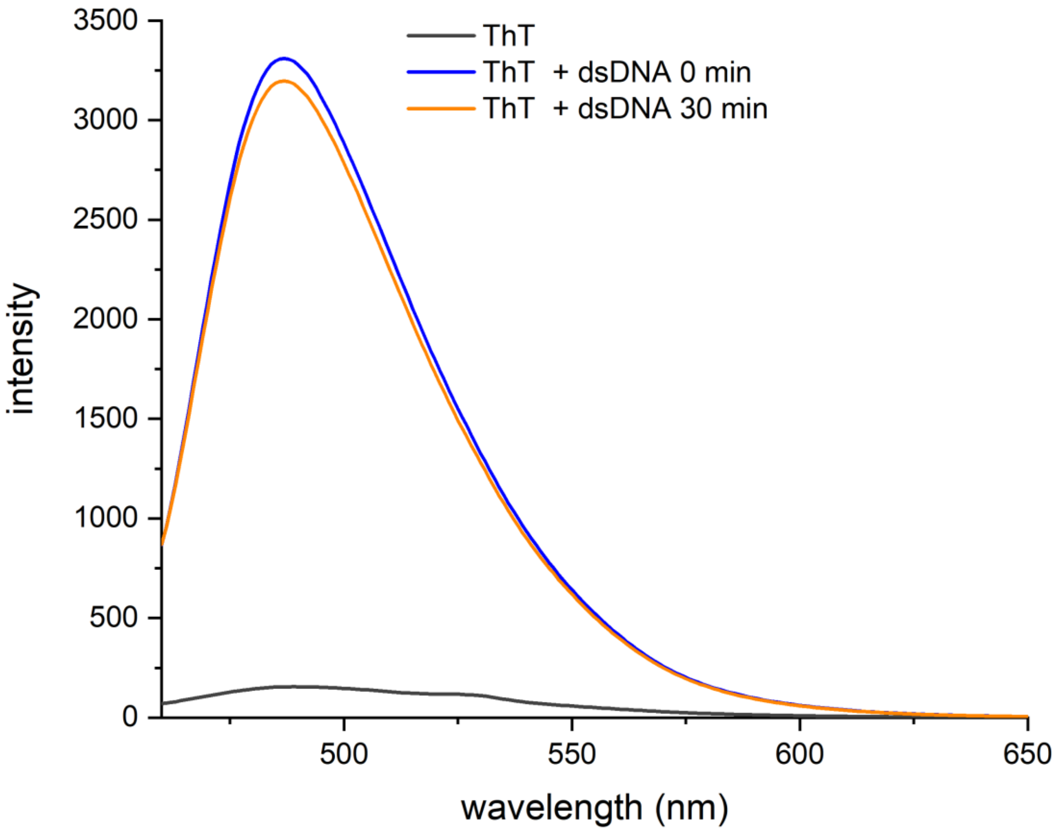

Figure S1. ThT fluorescence spectra in the absence and presence of dsDNA. Here, 1 ml of pure ThT solution (20 µM) with and without dsDNA (72 µM bp) was measured using a Jasco fluorospectrometer FP-8200 with an excitation wavelength of 450 nm. Directly after mixing ThT and dsDNA (0 min incubation), drastically enhanced fluorescence intensity is observed. The fluorescence intensity did not change significantly during 30 min incubation at room temperature.

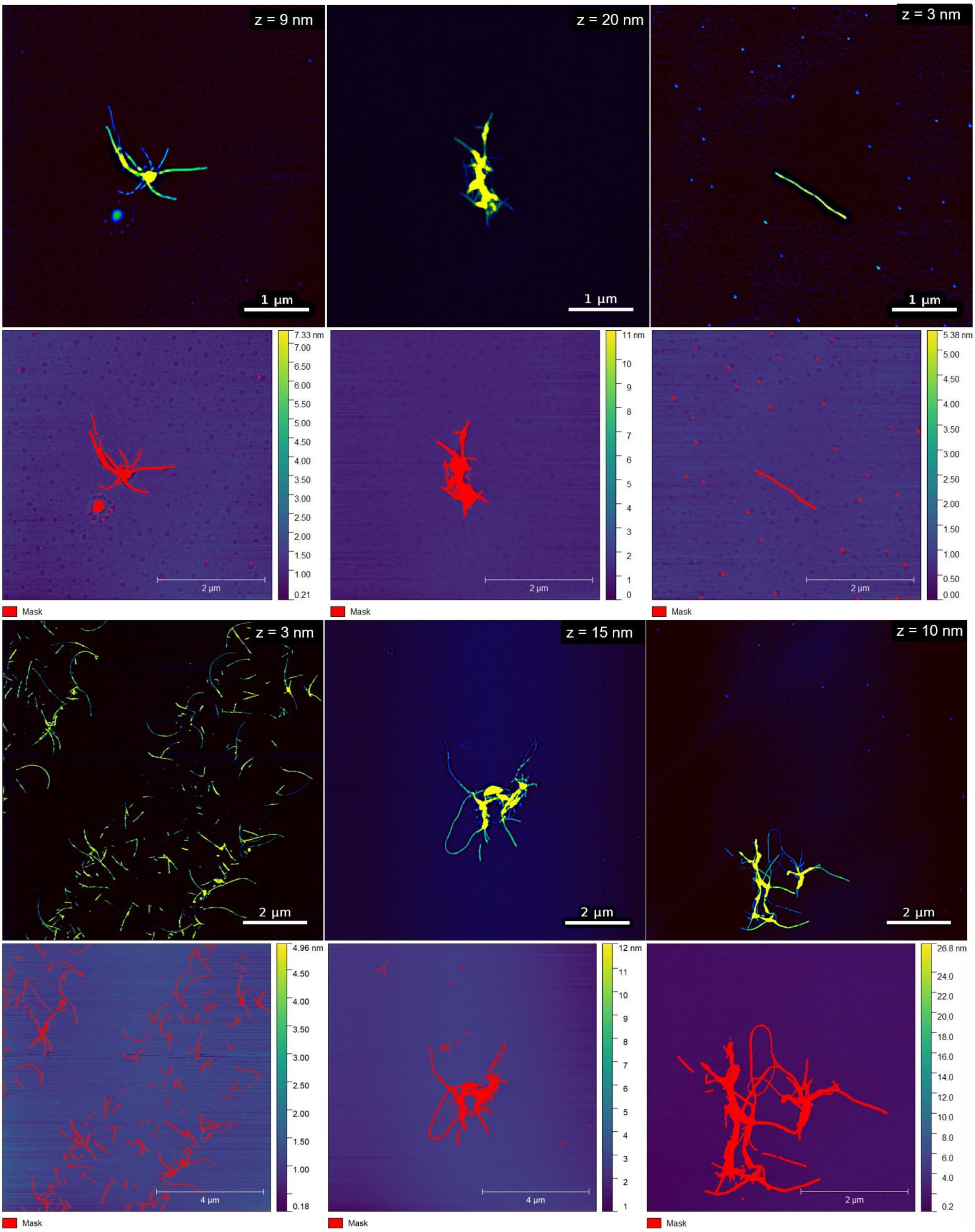

Figure S2. AFM images of pure hIAPP incubated without any DNA for 30 min. The ranges of the z-scales are given in the individual images. Below each image, the thresholded version of the original image used in the analysis of the morphological aggregate parameters is given with the analyzed aggregates highlighted in red.

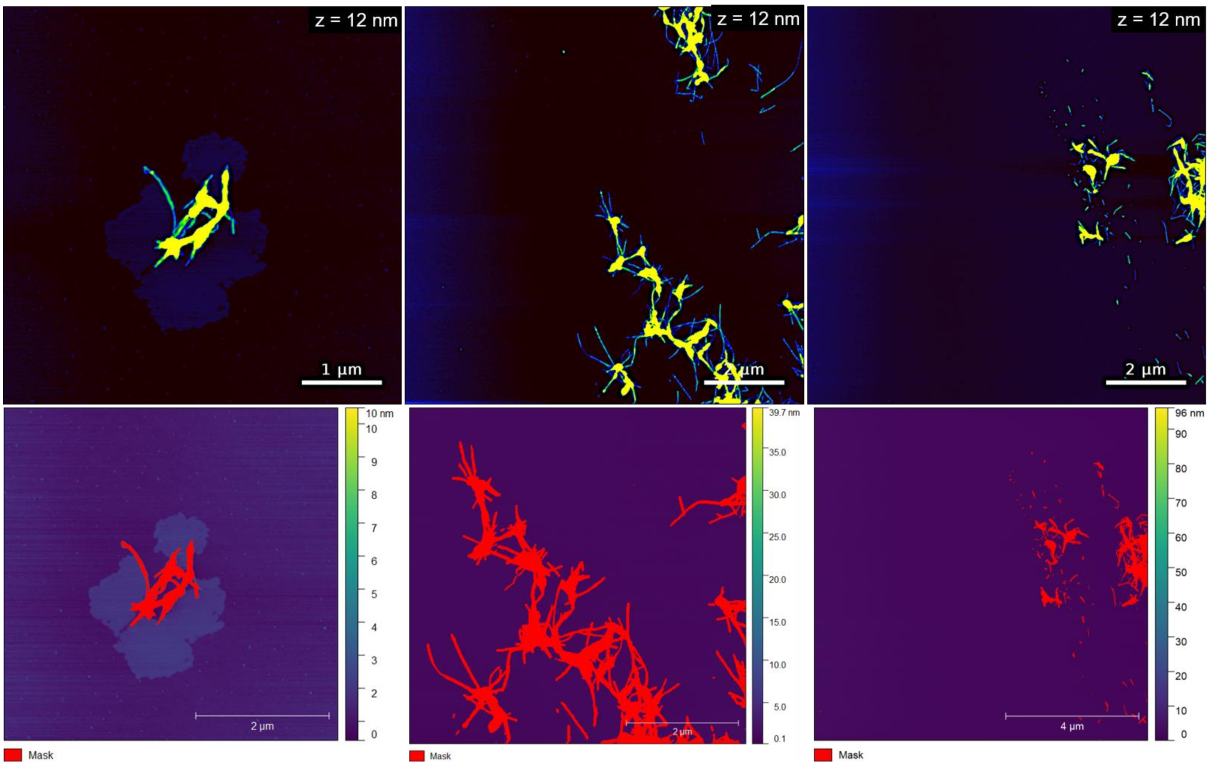

Figure S3. AFM images of pure hIAPP incubated without any DNA for 1 h. The ranges of the z-scales are given in the individual images. Below each image, the thresholded version of the original image used in the analysis of the morphological aggregate parameters is given with the analyzed aggregates highlighted in red.

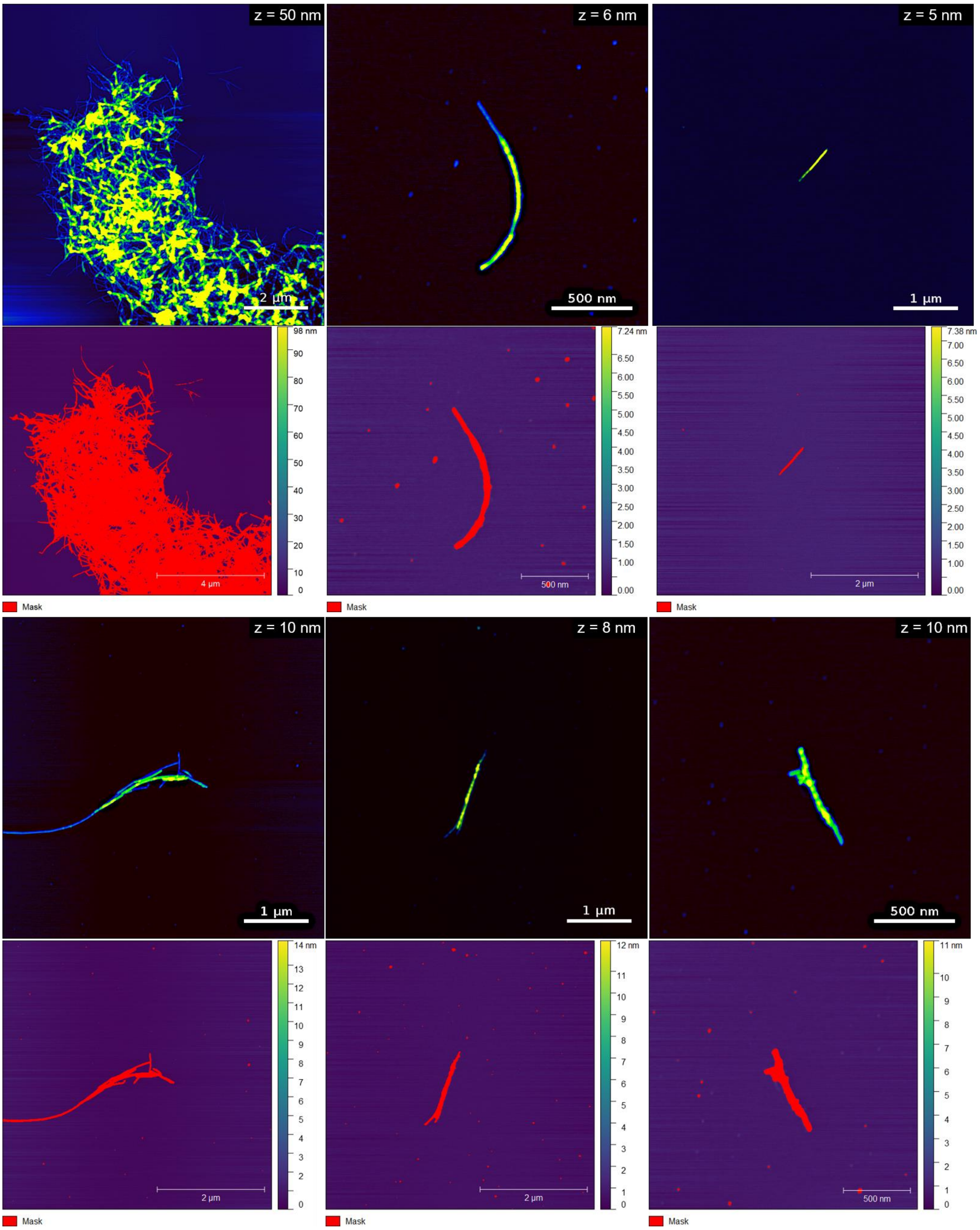

Figure S4. AFM images of pure hIAPP incubated without any DNA for 3 h. The ranges of the z-scales are given in the individual images. Below each image, the thresholded version of the original image used in the analysis of the morphological aggregate parameters is given with the analyzed aggregates highlighted in red.

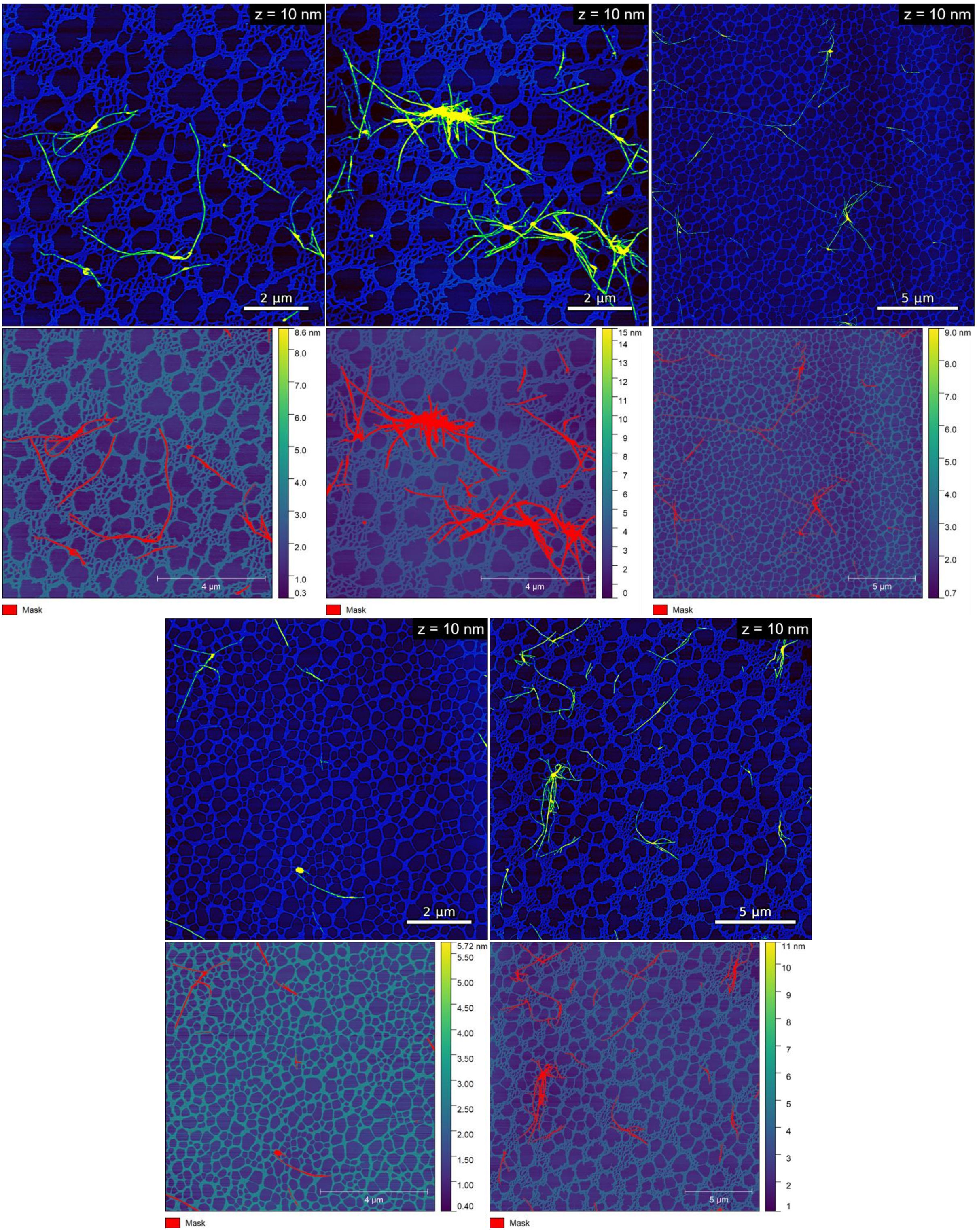

Figure S5. AFM images of hIAPP incubated with genomic dsDNA for 30 min. The ranges of the z-scales are given in the individual images. Below each image, the thresholded version of the original image used in the analysis of the morphological aggregate parameters is given with the analyzed aggregates highlighted in red.

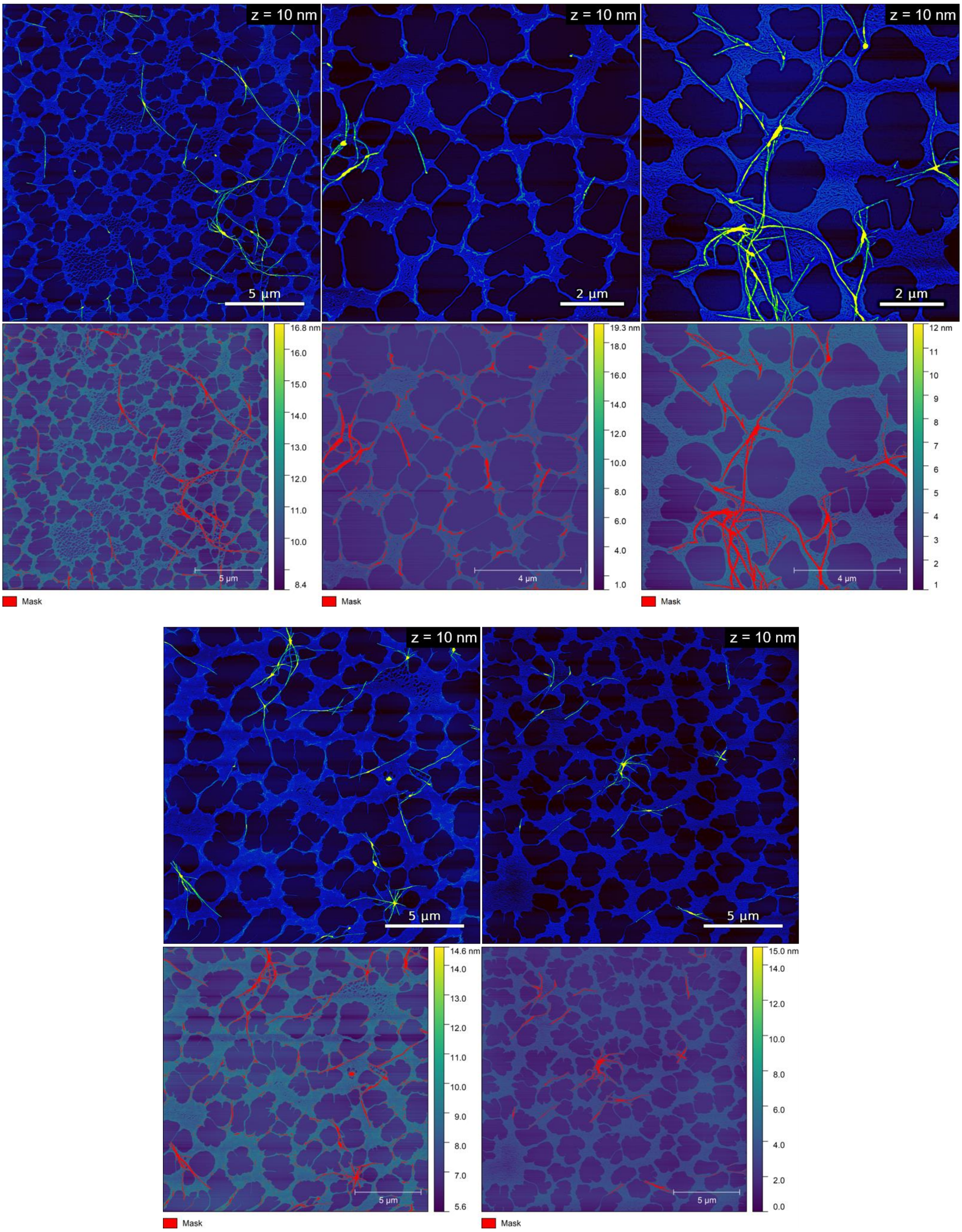

Figure S6. AFM images of hIAPP incubated with genomic dsDNA for 1 h. The ranges of the z-scales are given in the individual images. Below each image, the thresholded version of the original image used in the analysis of the morphological aggregate parameters is given with the analyzed aggregates highlighted in red.

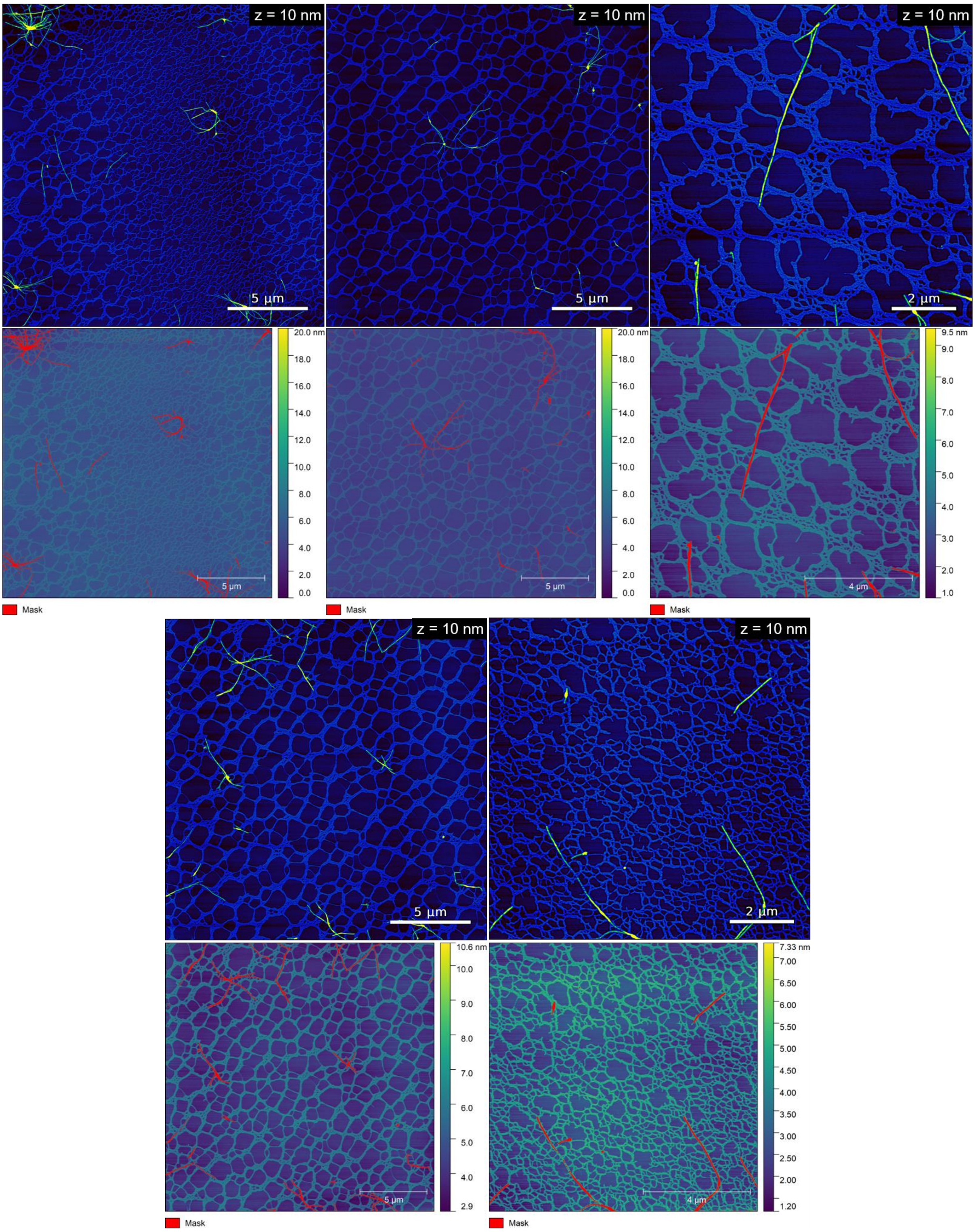

Figure S7. AFM images of hIAPP incubated with genomic dsDNA for 3 h. The ranges of the z-scales are given in the individual images. Below each image, the thresholded version of the original image used in the analysis of the morphological aggregate parameters is given with the analyzed aggregates highlighted in red.

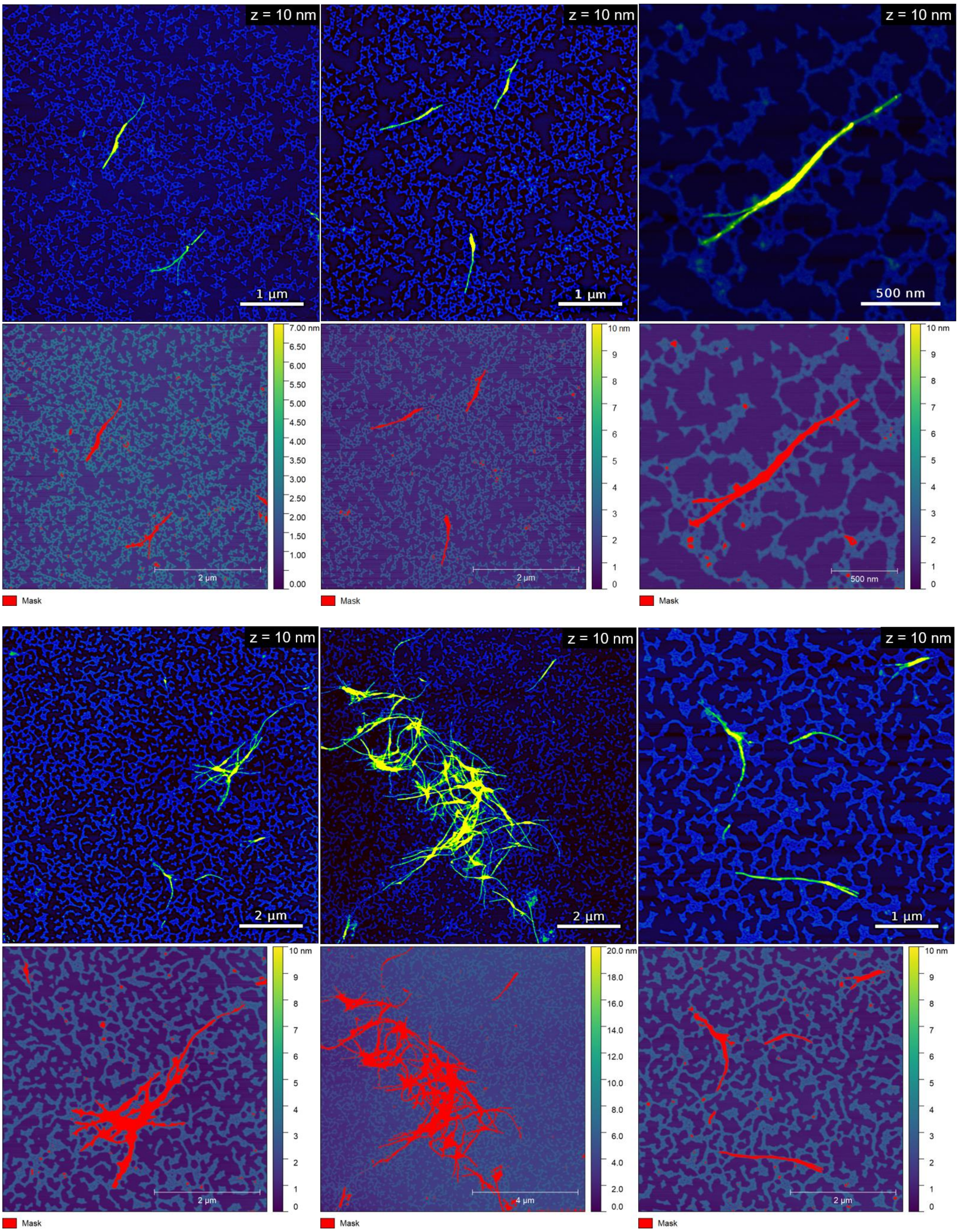

Figure S8. AFM images of hIAPP incubated with DNA origami triangles for 30 min. The ranges of the z-scales are given in the individual images. Below each image, the thresholded version of the original image used in the analysis of the morphological aggregate parameters is given with the analyzed aggregates highlighted in red.

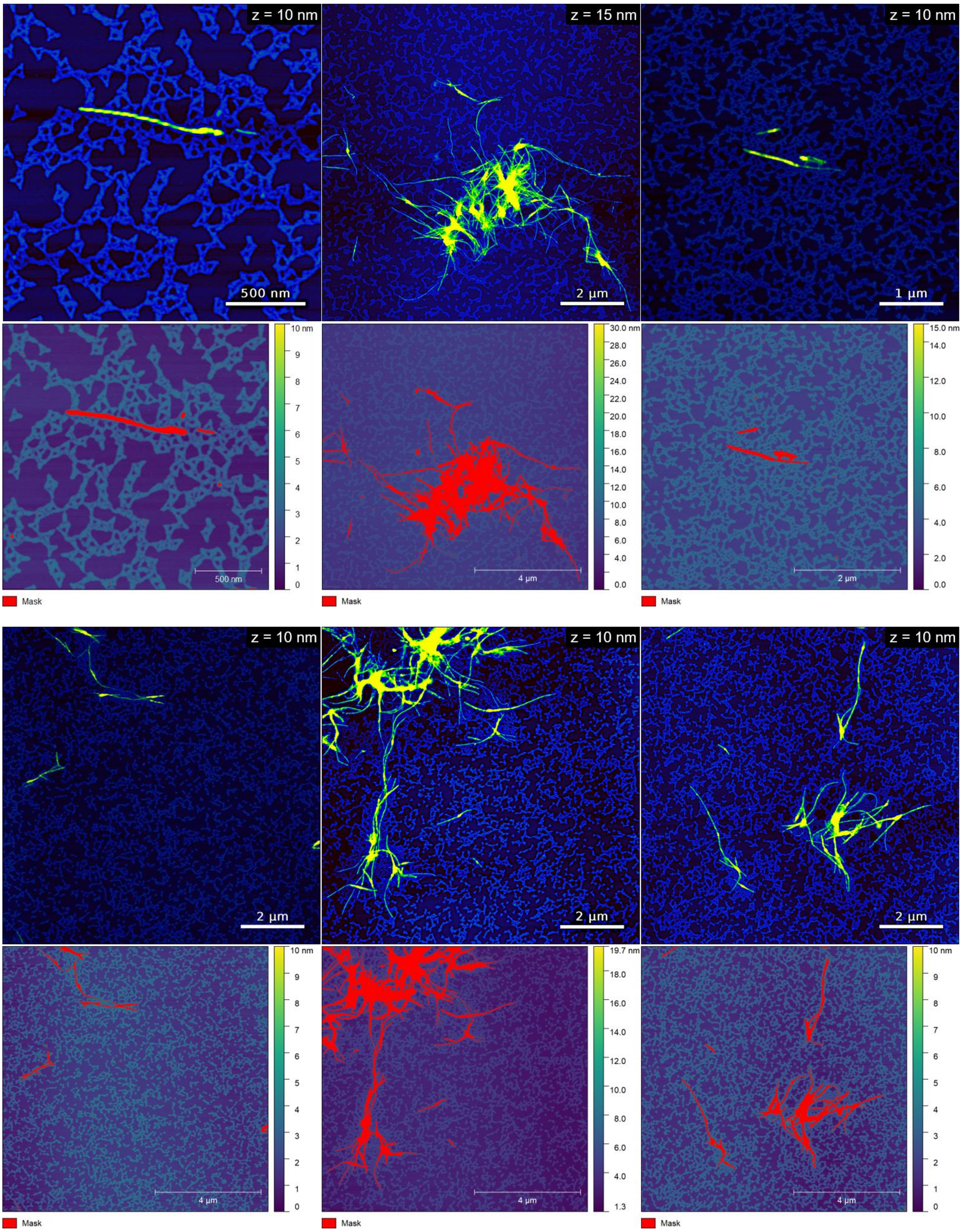

Figure S9. AFM images of hIAPP incubated with DNA origami triangles for 1 h. The ranges of the z-scales are given in the individual images. Below each image, the thresholded version of the original image used in the analysis of the morphological aggregate parameters is given with the analyzed aggregates highlighted in red.

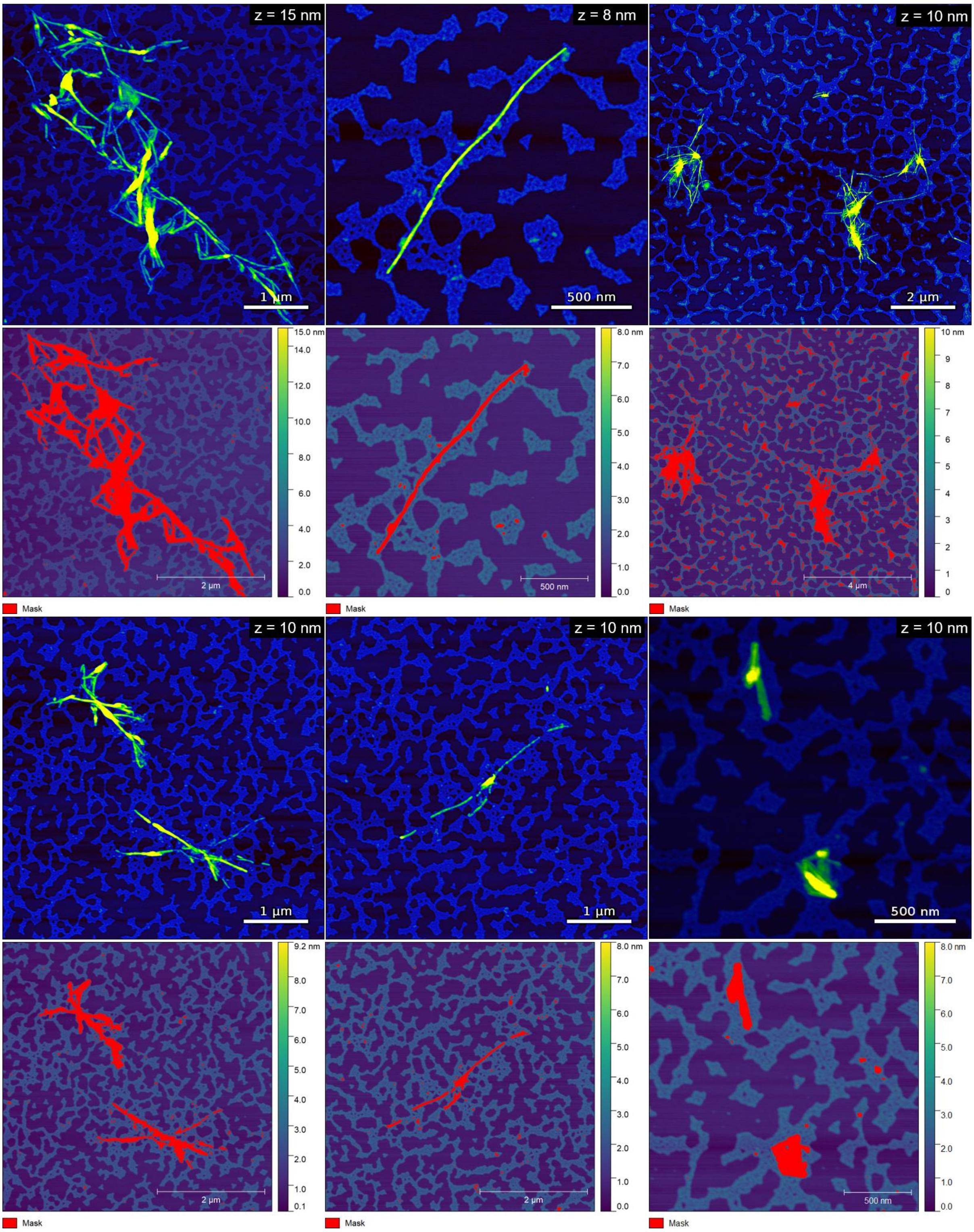

Figure S10. AFM images of hIAPP incubated with DNA origami triangles for 3 h. The ranges of the z-scales are given in the individual images. Below each image, the thresholded version of the original image used in the analysis of the morphological aggregate parameters is given with the analyzed aggregates highlighted in red.

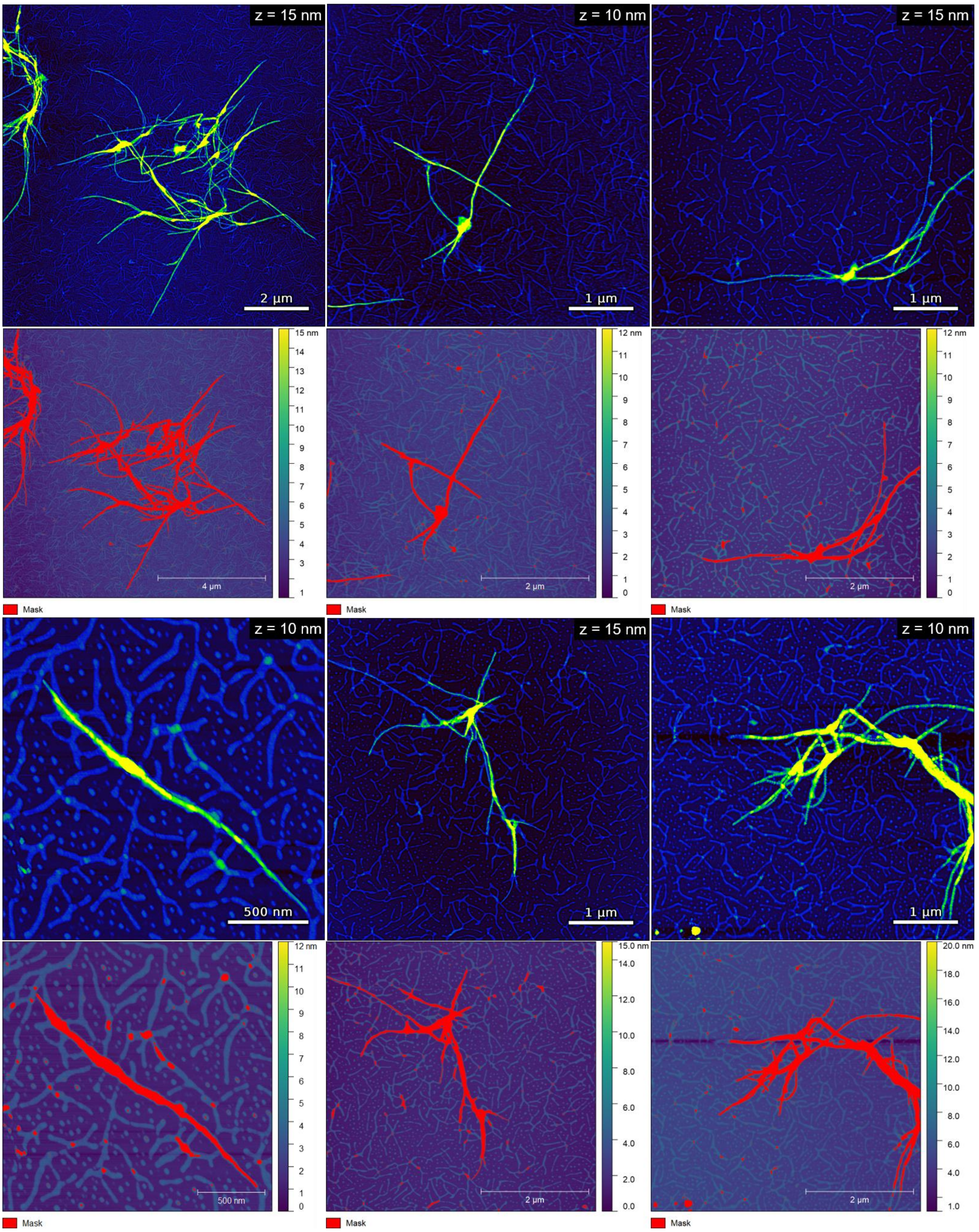

Figure S11. AFM images of hIAPP incubated with DNA origami 6HBs for 30 min. The ranges of the z-scales are given in the individual images. Below each image, the thresholded version of the original image used in the analysis of the morphological aggregate parameters is given with the analyzed aggregates highlighted in red.

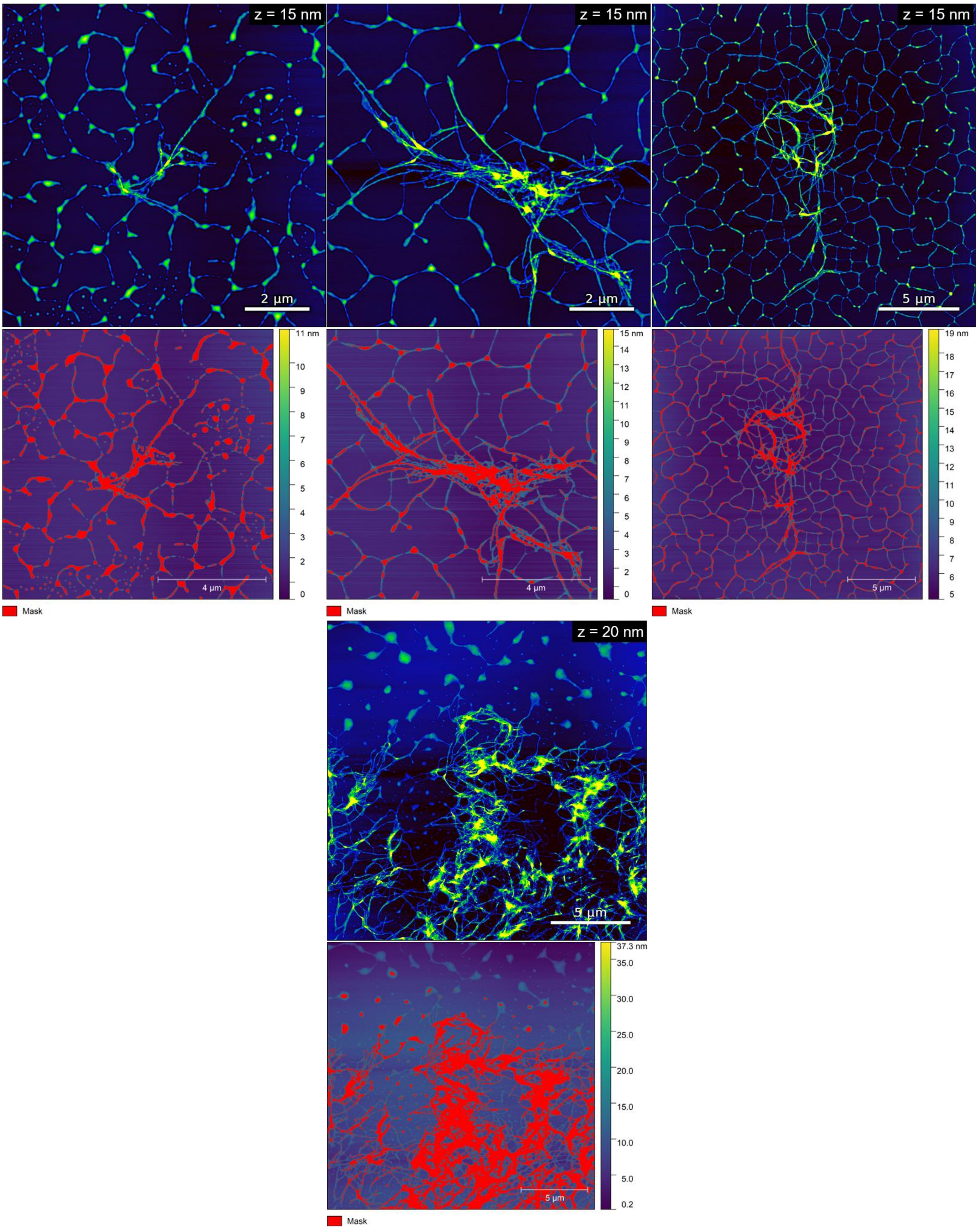

Figure S12. AFM images of hIAPP incubated with DNA origami 6HBs for 1 h. The ranges of the z-scales are given in the individual images. Below each image, the thresholded version of the original image used in the analysis of the morphological aggregate parameters is given with the analyzed aggregates highlighted in red.

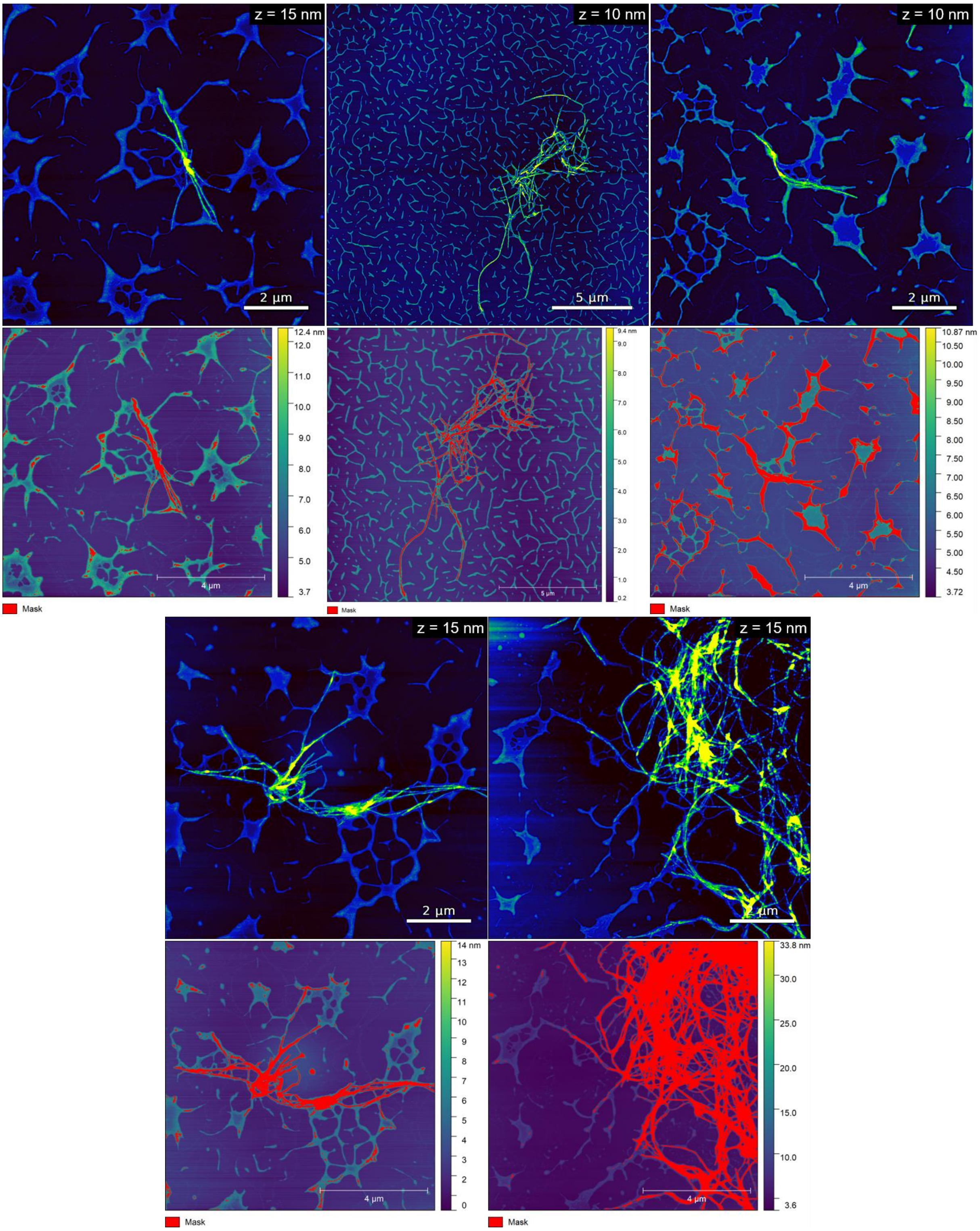

Figure S13. AFM images of hIAPP incubated with DNA origami 6HBs for 3 h. The ranges of the z-scales are given in the individual images. Below each image, the thresholded version of the original image used in the analysis of the morphological aggregate parameters is given with the analyzed aggregates highlighted in red.
